# Arthritis is associated with T-cell-induced upregulation of Toll-like receptor 3 on synovial fibroblasts

**DOI:** 10.1186/ar3384

**Published:** 2011-06-27

**Authors:** Wenhua Zhu, Liesu Meng, Congshan Jiang, Xiaojing He, Weikun Hou, Peng Xu, Heng Du, Rikard Holmdahl, Shemin Lu

**Affiliations:** 1 Department of Genetics and Molecular Biology, Xi'an Jiaotong University School of Medicine, Yanta West Road 76, Xi'an, Shaanxi 710061, People's Republic of China; 2 Key Laboratory of Environment and Genes Related to Diseases, Ministry of Education, Yanta West Road 76, Xi'an, Shaanxi 710061, People's Republic of China; 3 Department of Bone and Joint Diseases, Xi'an Red Cross Hospital, Nanguo Road 76, Xi'an, Shaanxi 710054, People's Republic of China; 4 Department of Orthopedics, First Affiliated Hospital, Xi'an Jiaotong University School of Medicine, Yanta West Road 277, Xi'an, Shaanxi 710061, People's Republic of China; 5 Division of Medical Inflammation Research, Department of Medical Biochemistry and Biophysics, Karolinska Institute, Alfred Nobels Allé 8, Huddinge, SE-171 77 Stockholm, Sweden; 6 Department of Epidemiology and Health Statistics, Xi'an Jiaotong University School of Medicine, Yanta West Road 76, Xi'an, Shaanxi 710061, People's Republic of China

## Abstract

**Introduction:**

Toll-like receptors (TLRs) are likely to play crucial roles in the pathogenesis of rheumatoid arthritis (RA). The aim of this study was to determine the key TLRs in synovium and explore their roles in the activation of fibroblast-like synoviocytes (FLSs) mediated by T cells in arthritis.

**Methods:**

Pristane-induced arthritis (PIA) was established by subcutaneous injection with pristane at the base of the rat's tail. TLR expression in synovium from PIA rats was detected at different time points by performing real-time PCR. Polyinosinic:polycytidylic acid (poly(I:C)) was intra-articularly administrated to PIA rats, and arthritis was monitored macroscopically and microscopically. Synovial TLR3 was detected by immunohistochemical staining. Rat FLSs were stimulated with pristane-primed T cells or pristane-primed, T-cell conditioned medium. The intervention of TLR3 in FLSs was achieved by specific short-hairpin RNA (shRNA) or an antibody. The migration ability of FLSs was measured by using the scratch test, and gene expression was detected by using real-time PCR. FLSs from RA patients were stimulated with various cytokines and TLR ligands, and TLR3 expression was detected by performing real-time PCR. In addition, with different concentrations of poly(I:C) stimulation, TLR3 expression of FLSs from RA patients and patients with osteoarthritis (OA) was compared.

**Results:**

Synovium TLR3 displayed early and persistent overexpression in PIA rats. TLR3 was expressed in FLSs, and local treatment with poly(I:C) synergistically aggravated the arthritis. Rat FLSs co-cultured with pristane-primed T cells showed strengthened migration ability and significant upregulation of TLR3, IFN-β, IL-6 and matrix metalloproteinase 3 (MMP3) expression, which could also be induced by pristane-primed, T-cell conditioned medium. The upregulation of cytokines and MMPs was blocked by shRNA or TLR3 antibodies. In RA FLSs with cytokine or TLR ligand stimulation, TLR3 expression exhibited remarkable upregulation. Furthermore, RA FLSs showed higher reactivity than OA FLSs to poly(I:C).

**Conclusions:**

TLR3 in the synovium of PIA rats was overexpressed, and activation of the TLR3 signaling pathway could aggravate this arthritis. The induction of TLR3 in FLSs resulted from T cell-derived inflammatory stimulation and could further mediate FLS activation in arthritis. We conclude that TLR3 upregulation of FLSs activated by T cells results in articular inflammation.

## Introduction

Rheumatoid arthritis (RA) is a chronic autoimmune disease characterized by synovial inflammation, cartilage and bone erosion and pannus formation [1,2]. Synovitis manifesting synoviocyte proliferation and activation has been considered the main cause of the secretion of proinflammatory cytokines and chemokines, the recruitment of inflammatory cells and the production of matrix metalloproteinases (MMPs) [3,4]. Accumulating evidence highlights a role of Toll-like receptors (TLRs) in mediating the synovial inflammatory response [5,6].

TLRs belong to the pattern recognition receptor family and connect innate and adaptive immunoresponses. Stimulation of TLRs with their ligands activates NF-κB, mitogen-activated protein kinase and IFN regulatory factor pathways [7]. A direct consequence of antigen-presenting cell activation by TLRs is to enhance the secretion of cytokines, as well as the upregulation of major histocompatibility complex (MHC) and costimulatory molecule expression, which facilitate the activation of adaptive immune responses [8].

It has been demonstrated that many TLRs are constitutively expressed in immune cells and synoviocytes. Previous studies have shown that TLR2, TLR3, TLR4 and TLR7 are overexpressed in the synovial tissue of RA patients [9-11] and that TLR2 and TLR4 expression in peripheral blood cells and macrophages from RA patients is also upregulated [12]. Interestingly, TLR ligands, such as peptidoglycan (PGN), CpG DNA, heat shock proteins and RNA from both infectious organisms and endogenous necrotic cells, have been identified in the joints of RA patients [13-15]. Such exogenous and endogenous TLR ligands have been shown to induce arthritis in mice upon intra-articular injection [16,17]. The synoviocytes activated by TLR ligands could produce proinflammatory cytokines and chemokines, such as TNF-α, IL-15, IFN-β, granulocyte chemotactic protein 2, RANTES (regulated on activation normal T-cell expressed and secreted) and monocyte chemotactic protein 2, which might contribute to synovitis maintenance and inflammatory cell infiltration [15,18-20]. Activated synoviocytes could also secrete MMPs, RANKL (receptor activator of NF-κB ligand) and vascular endothelial growth factor, which are involved in the cartilage degradation, joint destruction and angiogenesis in joints [10,21,22]. Thus, TLRs may play a vital role in mediating the synovial inflammation in both RA and experimental arthritis [23]. However, the relative role of the different TLRs in mediating and regulating arthritis is still unclear.

In a previous study, we found that TLR3 is the earliest and most prominently upregulated TLR in splenic macrophages by screening the TLR expression profile in pristane-induced arthritis (PIA), a MHC class II-restricted and T-cell-dependent arthritis rat model, and that downregulation of TLR3 expression modulates the severity of arthritis [24]. These findings regarding TLR3 provide an explanation for the initiation of the inflammatory response in immune organs, but the roles of TLRs in local inflammation of joints are still unclear. We hypothesize that a TLR may be induced by arthritogenic T cells and then mediates the activation of synoviocytes in local inflammatory responses. The aim of the present study was to answer the question which TLR is probably the key TLR in synovium in arthritis, whether triggering of the key TLR directly affects arthritis severity and how the key TLR in synoviocytes is induced to mediate the local inflammatory response.

## Materials and methods

### Rats

Dark Agouti (DA rats were bred in a specific pathogen-free animal house at the Department of Genetics and Molecular Biology, Xi'an Jiaotong University School of Medicine, Shaanxi, People's Republic of China. Age- and sex-matched rats were used in all experiments, and each group contained 8 to 10 rats 8 to 12 weeks old. The experiments were approved by the Institutional Animal Ethics Committee of the university.

### Arthritis induction and evaluation

Arthritis was induced by a single subcutaneous injection of 300 μL of pristane (ACROS Organics, Morris Plains, NJ, USA) at the base of the rat's tail. Arthritis development and severity were monitored by the change in the perimeter of the ankle and midpaw and assessed using a macroscopic scoring system as described previously [25].

For pathological examination, ankle joints of rats were sectioned and stained with H & E. The pathological severity of synovitis was estimated on the basis of four items: (1) the thickness of the synovium lining layer, (2) pannus, (3) synovium inflammatory cells and (4) angiogenesis. Each pathological item was scored on a scale ranging from 0 (normal) to 3 (most severe). Finally, synovitis was estimated by adding the scores on items one through four, and the maximum histopathological score was 12 for each ankle.

### RNA quantitation

Rats were killed at day 0 (naive rats, D0), day 6 (D6), day 12 (D12) or day 26 (D26) after pristane injection, and the synovium was collected for TLR expression quantitation. Total RNA was isolated using TRIzol reagent (Invitrogen, Carlsbad, CA, USA), and cDNA was synthesized by using the First Strand cDNA Synthesis Kit (Fermentas, Burlington, ON, Canada). Real-time PCR was performed by using iQ5 optical system software (Bio-Rad Laboratories, Hercules, CA, USA) with SYBR *Premix Ex Taq*™ II (TaKaRa, Ohtsu, Shiga, Japan) for TLR and cytokine mRNA quantitation. Relative gene expression normalized by β-actin was calculated by using the 2^-ΔΔ*C*t ^method. Information regarding primers, products and annealing temperatures is given in Table 1.

**Table 1:** Primer information for real-time PCR

Gene accession number	Sequence (5'-3')		Size, bp	Annealing temperature, °C
*tlr1 *(rat) [NM_001172120]	Forward	CAGCAGCCTCAAGCATGTCTA	82	60
	Reverse	CAGCCCTAAGACAACAATACAATAGAAGA		
*tlr2 *(rat) [NM_198769]	Forward	CTCCTGTGAACTCCTGTCCTT	74	60
	Reverse	AGCTGTCTGGCCAGTCAAC		
*tlr3 *(rat) [NM_198791]	Forward	GATTGGCAAGTTATTCGTC	205	54
	Reverse	GCGGAGGCTGTTGTAGG		
*tlr4 *(rat) [NM_019178]	Forward	GATTGCTCAGACATGGCAGTTTC	135	54
	Reverse	CACTCGAGGTAGGTGTTTCTGCTAA		
*tlr5 *(rat) [NM_001145828]	Forward	GGGCAGCAGAAAGACGGTAT	61	60
	Reverse	CAGGCACCAGCCATCCTTAA		
*tlr6 (rat)* [NM_207604]	Forward	AGAACCTTACTCATGTCCCAAAAGAC	79	60
	Reverse	AGATCAGATATGGAGTTTTGAGACAGACT		
*tlr7 *(rat) [NM_001097582]	Forward	GTTTTACGTCTACACAGTAACTCTCTTCA	75	60
	Reverse	TTCCTGGAGGTTGCTCATGTTTT		
*tlr8 *(rat) [NM_001101009]	Forward	GGGGTAACACACCGTCTA	150	60
	Reverse	GTCAAGGCGATTTCCACT		
*tlr9 *(rat) [NM_198131]	Forward	CCGAAGACCTAGCCAACCT	70	60
	Reverse	TGATCACAGCGACGGCAATT		
*ifn-β *(rat) [NM_019127]	Forward	CTTGGGTGACATCCACGACTAC	92	54
	Reverse	GGCATAGCTGTTGTACTTCTTGTCTT		
*il-6 *(rat) [NM_012589]	Forward	AAGAAAGACAAAGCCAGAGTC	263	60
	Reverse	CACAAACTGATATGCTTAGGC		
*mmp3*(rat) [NM_133523]	Forward	ATCCCCTGATGTCCTCG	147	54
	Reverse	TTTCGCCAAAAGTGCC		
*mmp13 *(rat) [NM_133530]	Forward	TTCAACCCTGTTTACCT	293	54
	Reverse	TTCTTTTTCCTTGTCCC		
*β-actin *(rat) [NM_031144]	Forward	GAGGGAAATCGTGCGTGAC	157	60
	Reverse	GCATCGGAACCGCTCATT		
*TLR3 *(human) [NM_003265]	Forward	AGCCTTCAACGACTGATGCT	201	60
	Reverse	TTTCCAGAGCCGTGCTAAGT		
*β- ACTIN *(human) [NM_001101]	Forward	AGTTGCGTTACACCCTTTCTTG	150	60
	Reverse	TCACCTTCACCGTTCCAGTTT		

### Immunohistochemical staining

The paraffin-embedded ankle joints from each rat were sectioned, and endogenous peroxidase was blocked with 0.3% H_2_O_2 _for 10 minutes. Each section was treated with 10 M urea and Antigen Repair solution I (Wuhan Boster Biological Technology, Ltd., Wuhan, China) for antigen repair and blocked with 1% bovine serum albumin. Then the slide was incubated with an anti-TLR3 polyclonal antibody (1:100 dilution; Santa Cruz Biotechnology, Santa Cruz, CA, USA) or control (rabbit immunoglobulin G (IgG)) overnight at 4°C, and the SABC Kit (Wuhan Boster Biological Technology, Ltd., Wuhan, China) was used for signal amplification and visualization according to the manufacturer's instructions. All sections were stained with 3,3'-diaminobenzidine and counterstained with hematoxylin.

### Administration of TLR ligands to PIA rats

Sixteen DA rats were used for polyinosinic:polycytidylic acid (poly(I:C)) administration experiments. Briefly, half of the rats were injected subcutaneously with 150 μL of pristane, and the other half were injected with normal saline (NS). At day 5 after pristane injection, 50 μg of poly(I:C) (Amersham Biosciences, Piscataway, NJ, USA) in 10 μL of NS were injected intra-articularly into one side ankle cavity of each rat in both groups, and the same volume of NS injected into the contralateral ankle served as a control [26]. The paws were divided into four groups: a pristane-poly(I:C) group (injection with systematic pristane and local poly(I:C)), a pristane-NS group, a NS-poly(I:C) group and a NS/NS group. Arthritis symptoms were evaluated in a blinded fashion every two to four days by using a macroscopic scoring system, and the perimeter changes in the ankles and midpaws were evaluated until D26. After the rats were killed, the synovium was collected for RNA quantitation. In another experiment, the joints were collected at D7 and D14 after pristane injection for pathological examination.

### Rat fibroblast-like synoviocyte and T-cell co-culture

Synovial fibroblasts from DA rats were isolated by collagenase digestion, cultured as described previously and used after passage 4 [27]. After four passages, the cells were morphologically homogeneous and exhibited the appearance of synovial fibroblasts, with a typical bipolar configuration visualized by inverse microscopy. The purity of the cells was detected by flow cytometric staining using antivimentin antibody [28], and the proportion of intracellular vimentin-positive cells was more than 95%.

Spleens from PIA rats and control rats were homogenized as a single-cell suspension, and red blood cells were lysed with 0.84% NH_4_Cl. Next, pristane-primed T cells and control T cells were isolated from the spleen single-cell suspensions by using the Nylon Fiber Column T (Wako Pure Chemical Industries Ltd., Osaka, Japan) as described previously [29]. All T cells were cultured in RPMI 1640 medium supplemented with 10% fetal bovine serum (FBS) and 3 μg/mL Concanavalin A (Con A; Sigma, St. Louis, MO, USA) for 72 hours before use.

Fibroblast-like synoviocytes (FLSs) were seeded into six-well plates at a density of 1 × 10^5 ^cells/well in DMEM supplemented with 10% FBS for 24 hours, then the medium was replaced and pristane-primed and control T cells activated by Con A were added at FLS:T cell ratios 5:1, 1:1, 1:5 and 1:10. After 24 hours of co-culturing, suspended T cells were washed with PBS and the remaining FLSs were used to isolate RNA for gene expression study.

### Rat FLS migration ability assay

Rat FLSs were plated for 24 hours as described above, and a 100-μL tip (Axygen Scientific, Inc., Union City, CA, USA) was used to draw a streak uniformly in each well, and the initial width (0 hour) of the streak was measured using Image-Pro Plus software (Media Cybernetics, Silver Spring, USA) and an inverted microscope (Olympus Co., Tokyo, Japan). Next, FLSs were co-cultured with pristane-primed and control T cells as ratios of 1:1 and 1:5, respectively, and the widths of the streaks were measured after 24 and 48 hours of co-culturing. FLS migration ability was calculated using the formula[(the initial width)-(the width after 24 or 48 hours of co-culturing)]/(the initial width)*100%.

### Rat FLS treatment with T-cell conditioned medium

Pristane-primed T cells and control T cells were isolated and cultured with Con A activation for 72 hours as described above, then the supernatant of the culture medium was collected and filtered through a 0.45-μm filter (Millipore, Eschborn, Germany). Rat FLSs were plated and incubated with pristane-primed T-cell or control T-cell conditioned medium for 24 hours, and TLR3, cytokine and MMP expression of FLSs was detected by performing real-time PCR.

### Intervention of TLR3 in rat FLSs

The pGC Silencer™ U6-Neo-GFP-RNAi plasmid (Genechem, Shanghai, China) containing the target sequence of the rat *tlr3 *gene (ACC TCG ACC TCA CAG AGA A as TLR3 shRNA) was used for TLR3 interference, and the sequence of the negative control shRNA (NC-shRNA) was TTC TCC GAA CGT GTC ACG T [24]. Briefly, FLSs were seeded into six-well plates at a density of 1 × 10^5 ^cells/well and transfected with the plasmids plus Lipofectamine™2000 reagent (Invitrogen, Carlsbad, CA, USA). TLR3 expression in FLSs was detected after 24-hour transfection for the knockdown efficiency assay. In T-cell co-culture experiments, TLR3-knocked-down FLSs were co-cultured with pristane-primed T cells at a ratio of 1:10, and the cytokine and MMP expression of FLSs was detected by performing real-time PCR after 24 hours. In a T-cell conditioned medium stimulation experiment, TLR3-knocked-down FLSs were incubated with pristane-primed T-cell conditioned medium for 24 hours, and the gene expression of FLSs was detected by real-time PCR.

In a TLR3 blockade assay, FLSs were seeded into six-well plates at a density of 1 × 10^5 ^cells/well. FLSs were treated with anti-TLR3 or isotype control antibodies at the concentration of 20 μg/mL for 90 minutes before co-culture with pristane-primed T cells at the ratio of 1:10 as described previously [24]. After 24-hour incubation, the gene expression of FLSs was detected by real-time PCR.

### Human synovial tissue preparation and FLS stimulation

Human synovial tissues were obtained from three RA patients and three osteoarthritis (OA) patients undergoing knee joint replacement surgery. All RA patients fulfilled the American College of Rheumatology criteria for the classification of RA [30]. OA was diagnosed according to the patients' clinical features. The Human Ethics Committee of Xi'an Jiaotong University approved this study, and the written, informed consent of all patients was obtained.

The FLSs from RA or OA patients were isolated as described previously [27]. FLSs were seeded into six-well plates at a density of 1 × 10^5 ^cells/well. After 24 hours, RA FLSs were stimulated with 10 ng/mL TNF-α, 1 ng/mL IL-1β, 1 ng/mL IFN-α, 1 ng/mL IFN-β, 10 μg/mL PGN, 10 μg/mL poly(I:C) and 10 ng/mL lipopolysaccharide (LPS), respectively. RA and OA FLSs were stimulated with 1, 10 and 100 μg/mL of poly(I:C). After 24-hour stimulation, cells were lysed with TRIzol reagent and TLR3 expression was detected by real-time PCR.

### Statistics

Quantitative data are expressed as means ± SEM. Statistical analysis was performed by using Student's *t*-test, the Mann-Whitney *U *test or two-way analysis of variance. Correlation analysis was performed by using Spearman's σ test. *P *< 0.05 was regarded as statistically significant.

## Results

TLR3 expression of synovium exhibited a remarkable increase in the PIA rat model

**Figure 1:**
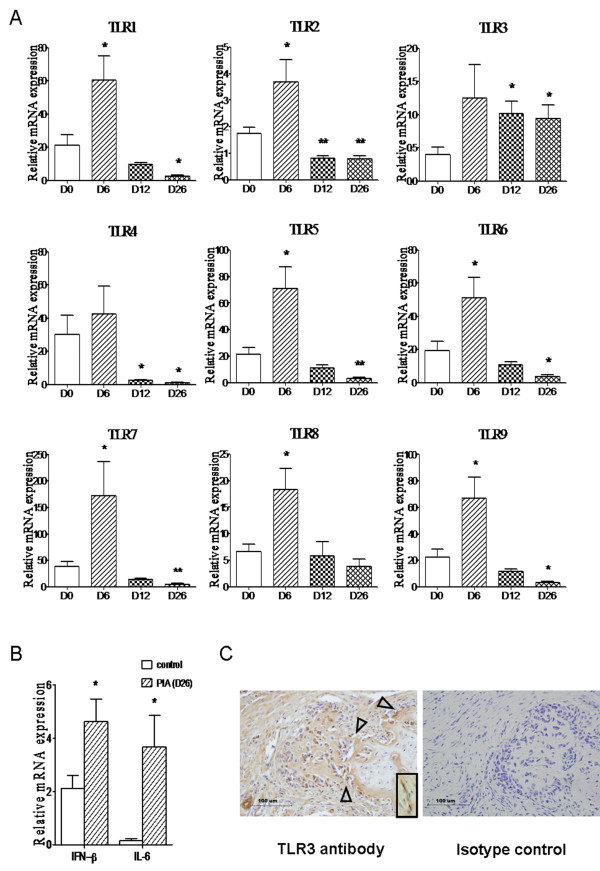
**Expression of Toll-like receptors in synovium from Dark Agouti rats with pristane-induced arthritis**. **(A) **Expression of TLR1 through TLR9 at D0, D6, D12 and D26, and **(B) **expression of IFN-β and IL-6 at D0 and D26 in synovium from rats (*n *= 8 to 10 for each time point) after pristane injection were measured by performing real-time PCR. Relative mRNA expression was compared with β-actin. Values shown are means ± SEM. Levels of significance between the pristane treatment group (D6, D12 and D26) and the control group (D0) were calculated by using Student's *t*-test (*P < 0.05, **P < 0.01). **(C) **TLR3 protein was assessed by performing immunohistochemistry in pristane-induced arthritis (PIA) synovium. TLR3 was strongly stained (brown, left-hand image) in synovium, especially in fibroblast-like synoviocytes (FLSs) (inset). FLSs from PIA joints expressed TLR3 predominantly at sites of attachment and invasion into cartilage and bone (open arrowheads). Staining specificity was assessed using isotype-matched antibodies as negative controls (right-hand image). Scale bars, 100 μm.

Arthritis-susceptible DA rats were given a single subcutaneous injection of pristane to induce arthritis. To find out the candidate key TLR, we analyzed TLR1 through TLR9 mRNA expression in the synovium of PIA rats at different time points, including D0 (normal phase), D6 (prearthritis phase), D12 (arthritis onset phase) and D26 (acute arthritis phase). Real-time PCR analysis confirmed significant upregulation of TLR1, TLR2 and TLR5 through TLR9 and, to a lesser extent, upregulation of TLR3 and TLR4 at D6 (Figure 1A). However, only TLR3 mRNA expression was upregulated and remained at a high level at D12 and D26 (Figure 1A). In contrast, TLR2 and TLR4 mRNA expression were significantly decreased at D12 and D26 and TLR1, TLR5, TLR6, TLR7 and TLR9 expression showed significant decreases at D26 (Figure 1A). The mRNA expression of both IFN-β and IL-6, which could be regulated by TLR3, showed significant increases in the PIA group (D26) compared with the control group (Figure 1B).

Next, we localized TLR3 protein expression in the ankles of PIA rats by immunohistochemical staining, and strong TLR3 staining was observed at synoviocytes, especially at hyperplastic FLSs (Figure 1C). Obviously, FLSs from PIA joints expressed TLR3 predominantly at sites of attachment and invasion into cartilage and bone (Figure 1C).

### Poly(I:C) mediated by TLR3 aggravated the severity of PIA

**Figure 2:**
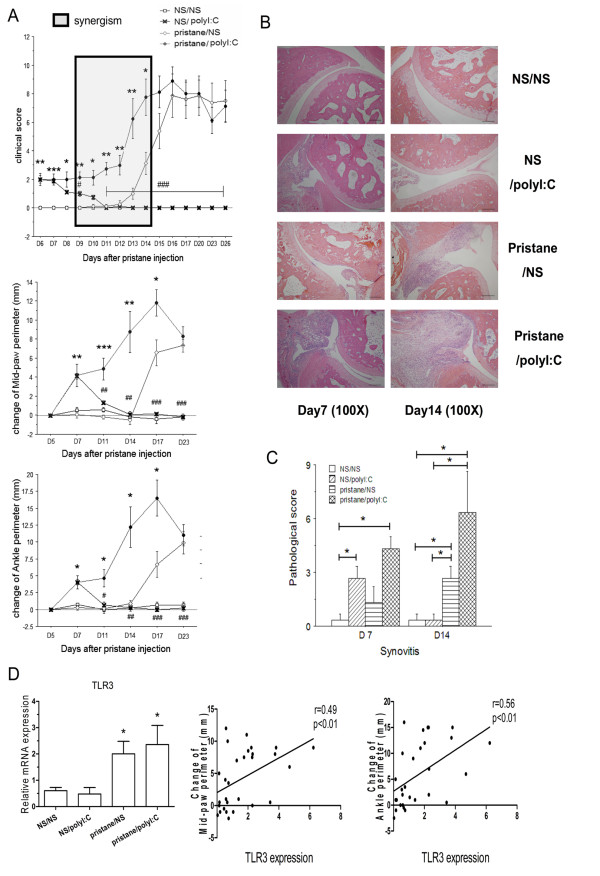
**More severity and increased histopathological scores of PIA caused by poly(I:C) local treatment**. **(A) **Poly(I:C) or NS was intra-articularly injected into different ankle cavities of each PIA rat and each control rat, and the arthritis clinical indexes, including clinical score, changes in midpaw perimeter and ankle perimeter of the paws, were compared among four groups (eight paws in each group). Clinical data were calculated using the Mann-Whitney *U *test (*/#*P *< 0.05, **/##*P *< 0.01, ***/###*P *< 0.001). *Pristane/poly(I:C) group vs. pristane/NS group. #Pristane/poly(I:C) group vs. NS/poly(I:C) group. Shaded box represents a synergistic effect assessed by two-way analysis of variance. **(B) **Representative histological images of ankle joints from each group at D7 and D14 are shown (H & E stain; original magnification, ×100). **(C) **Synovitis scores were quantified for histological analysis. Pathological data were calculated using the Mann-Whitney *U *test (*P < 0.05). *Comparison between groups as marked. **(D) **mRNA expression of TLR3 in synovium of each joint (eight joints in each group) at D26 was measured by real-time PCR. Values are presented as means ± SEM. Gene assay results were calculated by using Student's *t*-test (*P < 0.05). Correlation between TLR3 expression and the clinical indexes was measured by using Spearman's σ analysis.

Although the overexpression of TLR3 in the synovium of PIA rats was determined, whether the TLR3 signaling pathway is involved in the synovial inflammation of PIA was still undefined. So, the TLR3 ligand, which could trigger the activation of the TLR3 signaling pathway, was used locally to confirm the role of TLR3 in synovial inflammation. Poly(I:C) and NS were injected into different ankle cavities of each PIA rat and each control rat. The joints of rats in the NS/NS group did not show any signs of arthritis. Those of rats in the NS/poly(I:C) group exhibited mild swelling from D6 to D7, then it subsided gradually until disappearing at D11 (Figure 2A). The pristane/NS group joints developed arthritis similar to PIA, with onset beginning on D12, and the clinical score reached a peak at D16 and remained at a high level until D26. Interestingly, unlike those in the NS/poly(I:C) group, the joints of rats in the pristane/poly(I:C) group showed severe and stable swelling in ankles from D6, became increasingly aggravated and reached the most serious level at D14 (Figure 2A). The clinical score of rats in this group was significantly higher than that in the pristane/NS group from D6 to D14. Two-way analysis of variance confirmed a synergistic effect between the two factors, pristane and poly(I:C), from D9 to D14. The changes in the ankle and midpaw perimeters of each group displayed the same tendency as their clinical scores (Figure 2A). Compared with the pristane/NS group, the pristane/poly(I:C) group showed a remarkable change in ankle and midpaw perimeters from D7 to D17. Compared with the NS/poly(I:C) group, we also observed a significant change in ankle and midpaw perimeters from D11 to D23.

The ankle sections obtained at D7 and D14 were stained with H & E. The poly(I:C) injection groups, comprising the NS/poly(I:C) group and the pristane/poly(I:C) group, showed pathologic changes with abundant inflammatory cell infiltrates and synovium hyperplasia at D7. The pathologic changes in the NS/poly(I:C) group recovered at D14, but those in the pristane/NS group and the pristane/poly(I:C) group became aggravated with obvious bone and cartilage destruction, especially in the pristane/poly(I:C) group (Figure 2B). The pathological scores with regard to synovitis showed a significant difference between the NS/poly(I:C) group, the pristane/poly(I:C) group and the NS/NS group at D7 (Figure 2C). Not surprisingly, the pristane/poly(I:C) group and the pristane/NS group had higher synovitis scores at D14 compared with the NS/NS group and the NS/poly(I:C) group. The synovitis of the pristane/poly(I:C) group was more severe than that of the pristane/NS control group (Figure 2C).

Both the pristane/poly(I:C) group and the pristane/NS group had increased TLR3 mRNA expression in synovium at D26 compared with the NS/NS group (Figure 2D). In contrast, TLR3 expression did not differ between the NS/poly(I:C) group and the NS/NS group (Figure 2D). TLR3 mRNA expression correlated with the clinical indexes: the changes in ankle and midpaw perimeters (Figure 2D). On the basis of the ligand stimulation experiment, we concluded that overexpression of TLR3 in FLSs was involved in mediating arthritis development. However, the mechanism of the induction of TLR3 expression in FLSs was still unknown, so a co-culture of FLSs and splenic T cells was performed to investigate whether TLR3 might be induced by infiltrated T cells and then involved in synoviocyte activation.

### Rat FLSs were activated by pristane-primed T cells with increased TLR3 expression

**Figure 3:**
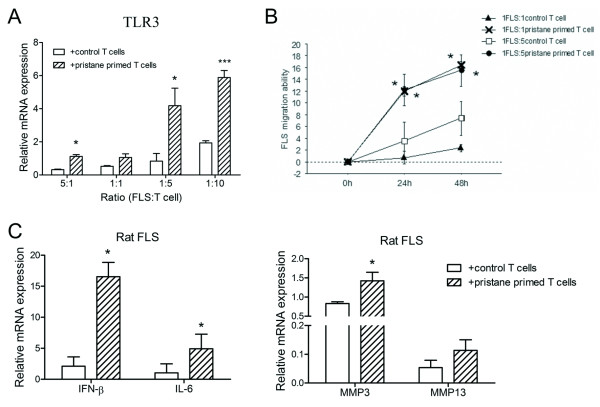
**Functional analysis of rat FLSs co-cultured with pristane-primed T cells**. Rat FLSs were co-cultured with pristane-primed or control T cells, and relative mRNA expression of genes in FLSs was detected by real-time PCR. **(A) **TLR3 mRNA expression in FLSs was measured 24 hours after co-culture with series FLS:T-cell ratios of 5:1, 1:1, 1:5 and 1:10. **(B) **FLS migration ability was analyzed 24 and 48 hours after co-culture with FLS:T-cell ratios of 1:1 and 1:5. The migration ability of FLSs co-cultured with pristane-primed T cells was compared to that co-cultured with control T cells. **(C) **Cytokine (IFN-β and IL-6) and matrix metalloproteinase MMP3 and MMP13 expression were detected 24 hours after co-culture with the cell ratio 1:10. Their expression in FLSs co-cultured with pristane-primed T cells was compared to that co-cultured with control T cells. Data are presented as means ± SEM of four replicated determinations from three independent experiments. Levels of significance were calculated by using Student's *t*-test (*P < 0.05, ***P < 0.001).

Rat FLSs were co-cultured with a series of pristane-primed or control T cells. TLR3 mRNA expression tended to be higher with increased T cells in both co-culture groups (Figure 3A). However, TLR3 expression of FLSs stimulated with pristane-primed T cells showed a higher increase compared with FLSs stimulated with control T cells at co-culture ratios of 5:1, 1:5 and 1:10 (Figure 3A). In addition, it seemed irregular that other TLRs were expressed at different co-culture ratios (Additional file 1). At the ratio of 5:1, TLR4 and TLR6 expression were increased in the pristane-primed T cell group. With the co-culture ratio at 1:1, TLR2, TLR4, TLR6, TLR8 and TLR9 expression levels were decreased significantly. Interestingly, except for TLR3 at the ratio of 1:5, other TLR expression levels were not upregulated. With the maximum co-culture ratio at 1:10, nearly all TLRs (except TLR1) were induced. Comparing all TLR expression regulation, we considered that TLR3 in FLSs should be more important and more specific with pristane-primed T-cell stimulation.

After being exposed to pristane-primed T cells for 24 and 48 hours, FLSs also showed enhanced migration ability when the co-culture ratios of FLSs and T cells were 1:1 and 1:5 (Figure 3B). Coincidentally, IFN-β, IL-6 and MMP3 expression of FLSs, which were stimulated with pristane-primed T cells for 24 hours, showed significantly high levels compared with those stimulated with control T cells, whereas MMP13 expression showed a trend toward upregulation (Figure 3C). Obviously, TLR3 expression in FLSs was induced by pristane-primed T-cell stimulation, and the FLSs were activated with more cytokine and MMP secretion. Whether synoviocyte activation was mediated by the TLR3 signaling pathway needed to be confirmed by conducting a TLR3 intervention experiment.

### Intervention of TLR3 blocked the activation of FLSs stimulated by pristane-primed T cells

**Figure 4:**
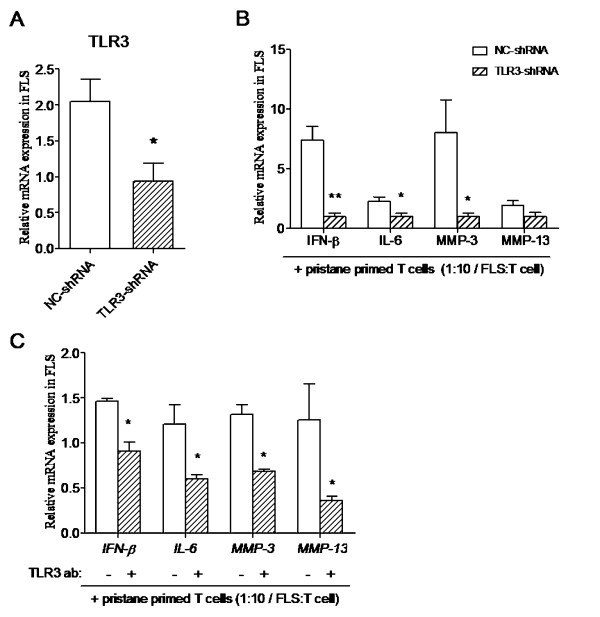
**Blockade of T-cell-activated FLSs via TLR3 signaling pathway intervention**. Rat FLSs were transfected with TLR3-shRNA plasmid and a negative control. **(A) **TLR3 mRNA expression was detected 24 hours after transfection. TLR3-shRNA- and NC-shRNA plasmid-transfected FLSs were co-cultured with pristane-primed T cells, and **(B) **cytokine (IFN-β and IL-6) and MMP3 and MMP13 expression were detected at 24 hours. **(C) **Rat FLSs were preincubated with TLR3 antibody and isotype IgG, followed by pristane-primed T-cell co-culture for 24 hours and then expression of IFN-β, IL-6, MMP3 and MMP13 was detected. Data are presented as means ± SEM of four replicated determinations from three independent experiments. Levels of significance were calculated by using Student's *t*-test (*P < 0.05, **P < 0.01).

First, RNA interference (RNAi) targeting rat TLR3 was used to interfere with TLR3 expression in FLSs. After 24-hour transfection, TLR3 expression was significantly downregulated in FLSs transfected with TLR3-shRNA plasmid (Figure 4A). Next, FLSs transfected with TLR3-shRNA and NC-shRNA plasmids were co-cultured with pristane-primed T cells at the ratio of 1:10 for 24 hours. IFN-β, IL-6 and MMP3 mRNA expression of FLSs was significantly reduced in the TLR3-shRNA groups compared with the NC-shRNA group, whereas MMP13 expression only displayed a tendency to decrease (Figure 4B).

Besides downregulation of TLR3 by RNAi, TLR3 antibody was used to block the TLR3 signaling pathway in rat FLSs. With TLR3 antibody or isotype IgG preincubation, rat FLSs were co-cultured with pristane-primed T cells at the ratio of 1:10 for 24 hours. All expression of IFN-β, IL-6, MMP3 and MMP13 was significantly reduced in the TLR3 antibody treatment group (Figure 4C).

### Soluble inflammatory factors from T cells activated FLSs via TLR3 in rats

**Figure 5:**
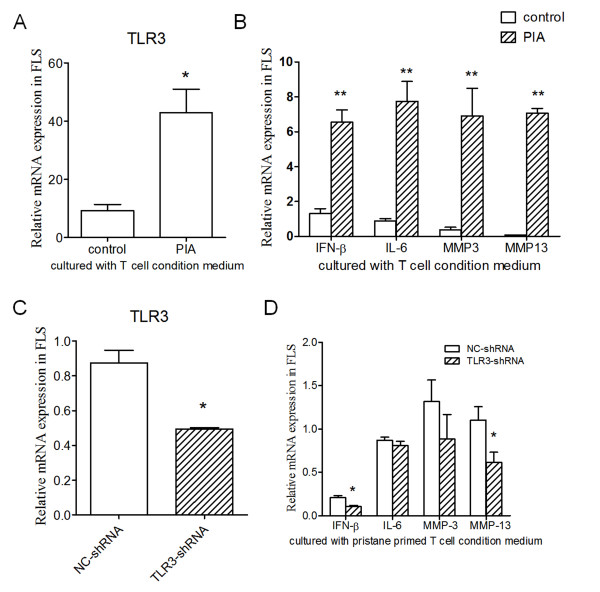
**Activation of FLSs via TLR3 induction stimulated by pristane-primed, T-cell-conditioned medium**. Rat FLSs were incubated with pristane-primed T-cell- or control T-cell-conditioned medium for 24 hours, and **(A) **TLR3 and **(B) **functional gene expression in FLSs were determined by using real-time PCR. Then rat FLSs were transfected with TLR3-shRNA plasmid and a negative control. **(C) **TLR3 mRNA expression was detected 24 hours after transfection. **(D) **TLR3-shRNA plasmid- and NC-shRNA plasmid-transfected FLSs were incubated with pristane-primed T cell condition medium, and the IFN-β, IL-6, MMP3 and MMP13 expression was detected at 24 hours. Data are presented as means ± SEM from three independent experiments. Levels of significance were calculated by using Student's *t*-test (*P < 0.05, **P < 0.01).

To investigate whether the induction of TLR3 was due to cell-cell interaction between FLSs and T cells or to the stimulation of soluble factors derived from T cells, we incubated rat FLSs with pristane-primed T-cell conditioned medium (PIA group) or control T-cell conditioned medium (control group), respectively, for 24 hours. TLR3 mRNA expression showed a significant upregulation in the PIA group (Figure 5A). Meanwhile, compared with the control group, the expression of IFN-β, IL-6, MMP3 and MMP13 was significantly induced in the PIA group (Figure 5B). Therefore, soluble stimulation might be a main cause of TLR3 induction in rat FLSs.

Next, we used TLR3-specific RNAi to interfere with TLR3 expression in FLSs. After 24-hour transfection, TLR3 expression showed significant downregulation in FLSs transfected with TLR3-shRNA plasmid (Figure 5C). Then FLSs transfected with TLR3-shRNA and NC-shRNA plasmids were incubated with pristane-primed T-cell conditioned medium for 24 hours. Compared with the NC-shRNA group, the results showed significant downregulation of IFN-β and MMP-13 and, to a lesser extent, MMP-3 in the TLR3-shRNA group (Figure 5D). However, IL-6 was not blocked by the TLR3 RNAi, suggesting that other regulation mechanisms might be present. Overall, the data confirmed that soluble inflammatory factors from T cells could be the main source driving the activation of FLSs via TLR3 in rats.

### TLR3 expression in FLSs from humans was induced by soluble factor stimulation

**Figure 6:**
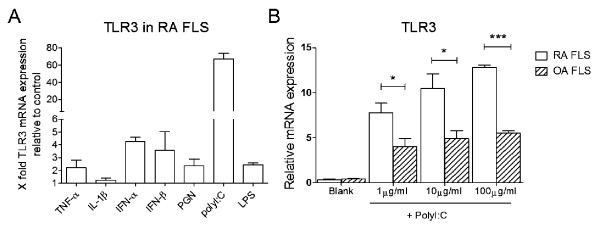
**Regulation of TLR3 expression in RA FLSs stimulation by various inflammatory factors**. **(A) **FLSs from RA patients (*n *= 3) were stimulated with TNF-α, IL-1β, IFN-α, IFN-β, PGN, poly(I:C) and LPS for 24 hours, and TLR3 expression was regulated to different extents. Values are mean ± SEM fold induction of TLR3 mRNA relative to unstimulated cells (set as control). Baseline expression (control group) was set at 1. **(B) **Induction of TLR3 mRNA expression in FLSs from RA patients (*n *= 3) and OA patients (*n *= 3) with series poly(I:C) dose stimulation. Data are presented as means ± SEM from three independent experiments. Levels of significance were calculated by using Student's *t*-test (*P < 0.05, ***P < 0.001).

Next, we collected the FLSs from RA and OA patients to test whether TLR3 could also be induced by soluble stimulation in the human system. RA FLSs stimulated with TNF-α, IFN-α, IFN-β, PGN, poly(I:C) and LPS showed remarkable elevation in TLR3 mRNA expression, especially with poly(I:C) (Figure 6A). FLSs stimulated with IL-1β showed only slightly higher TLR3 expression (Figure 6A). Then RA and OA FLSs were treated with 1, 10 and 100 μg/mL poly(I:C) stimulation for 24 hours. The more than four-passaged, purified FLSs from RA and OA patients showed no difference in TLR3 expression (Figure 6B). However, TLR3 expression in both groups was significantly induced by poly(I:C) and showed a tendency to increase with the series of poly(I:C) concentrations of RA but not OA FLSs. Moreover, TLR3 showed significantly higher expression in RA FLSs than in OA FLSs with poly(I:C) stimulation (Figure 6B).

## Discussion

Many TLRs in previous studies of RA have been reported to play a vital role in the pathogenesis of arthritis. However, which TLR is key to mediating the initiation and maintenance of joint inflammation is still unclear, owing to the limited number of patient samples. Accordingly, a single subcutaneous injection of pristane in DA rats led to chronic relapsing arthritis similar to RA in humans. We comparatively analyzed the expression of TLR1 through TLR9 in synovium during the whole course of arthritis initiation and development to investigate the roles of TLRs systematically and dynamically in a PIA model. Nearly all TLR expression showed a transient induction at the early stage (D6), at which point arthritis had not yet occurred. This finding is probably due to the general stress reaction stimulated by pristane, since we have observed that there is a systemic inflammatory response in spleen and lymph nodes with the activation of TLRs and the secretion of proinflammatory cytokines [24]. Therefore, more attention to the disease onset and development phases should be a priority. Surprisingly, we have demonstrated in the present study that a compelling TLR is TLR3, which has early and stable overexpression in synovium exclusively at initiation and development stages of arthritis. Coincidentally, in the PIA rat model, IFN-β, a TLR3-related cytokine, and IL-6 showed significant increases at D26. TLR3 has been reported to be expressed mainly on dendritic cells and fibroblasts, as well as on murine macrophages [31]. In the synovium of RA patients, it has been found that most of the synoviocytes that expressed TLR3 are synovial fibroblasts, except macrophages [10,15]. Consistent with previous observations in humans, TLR3 protein expression was detected by immunohistochemical staining in FLSs of PIA rats, suggesting that FLSs constitute a major cellular source of TLR3 production in inflamed synovial tissue. In other words, the hyperplasia and activation of FLSs might be mediated by TLR3 signaling. One possibility is that upregulated TLR3 could recognize the RNA released from necrotic synovial fluid cells and then activate RA synovial fibroblasts to promote cytokine secretion and expedite osteoclastogenesis [15,32].

According to the above findings, we suggest that TLR3 in FLSs may play an important role in the local inflammation of PIA. In a previous study, intra-articular treatment with poly(I:C) was found to cause a rapid and strong synovitis in rats [26], which suggests the involvement of TLR3 signaling in inflammation mediation. To confirm that overexpression of TLR3 in FLSs is functional in joint inflammation induced by pristane, we administrated poly(I:C) locally to PIA rats and observed the synergistic effect. The results showed that PIA rats intra-articularly treated with poly(I:C) compared with rats treated with poly(I:C) alone exhibited more persistent inflammation and joint destruction, which might be due to the upregulation of TLR3 expression in PIA. Meanwhile, comparing the PIA group and the PIA/poly(I:C) group, we found that treatment with poly(I:C) could exacerbate arthritis, especially at the initial stage of arthritis, according to both the clinical and pathological results. Obviously, the above results confirm that there is a strong synergistic effect between pristane and poly(I:C). TLR3 expression was upregulated in both pristane treatment groups, especially in the pristane/poly(I:C) group, and significantly correlated with arthritis severity, suggesting that the activation of TLR3 on fibroblasts triggered by poly(I:C) causes more severe arthritis. These results validate the importance of TLR3 in PIA *in vivo*. However, the cause of the upregulation of TLR3 expression and the activation of FLSs in arthritis is not known yet.

Accumulating evidence favors the involvement of an adaptive autoimmune process in the etiopathogenesis of RA and experimental arthritis [33,34]. Recently, several models of spontaneous arthritis due to perturbations in the TCR signaling pathway have been identified, and the effector mechanisms in the model are T cell-mediated [35]. Moreover, the vast majority of lymphocytes infiltrating RA synovium are CD4^+ ^αβ T cells [36,37]. PIA is also a T-cell-dependent disease which can be transferred with CD4^+ ^αβ T cells [38]. T cells isolated from the spleens of PIA rats transfer severe arthritis, showing that the critical arthritogenic change induced by pristane is present in the spleen. Meanwhile, there is a selective accumulation of activated CD4^+ ^T cells in PIA synovial membrane. Administration of anti-CD4 antibody, but not anti-CD8 antibody, can ameliorate PIA (W Zhu, L Meng, J Tuncel, S Carlsen, S Lu and R Holmdahl. unpublished data). These data suggest that the splenic, pristane-primed, arthritogenic T cells, which specifically infiltrate into joints, might be the inflammatory trigger for FLS activation, joint destruction and arthritis chronicity. Thus, we co-cultured the splenic T cells with rat FLSs to investigate the intercellular interaction in synovial inflammation. It was a surprising finding that pristane-primed T cells significantly elevated TLR3 expression in FLSs and that FLSs also exhibited an enhanced migration ability and increased cytokine and MMP secretion. In other words, the activated T cells in the organs of the immune system led to the induction of FLSs in arthritic joints. Meanwhile, we found that other TLR expression was regulated erratically. With the exception of TLR3, TLRs were not induced significantly under the co-culture ratio of 1:5, suggesting that TLR3 might be preferentially induced with pristane-primed T cell-specific stimulation. With the maximum co-culture ratio (1:10), nearly all TLR (but not TLR1) expression was increased, which was probably due to the secondary inflammatory response. Taken together with the comprehensive analysis, we considered that TLR3 should be a more important and specific TLR in FLS induction and thus we gave priority attention to TLR3 in our following work, although we still could not rule out other TLR roles in joint inflammation, which need to be confirmed by further studies.

Intervention of both TLR3 expression and the TLR3 signaling pathway in FLSs blocked the upregulation of cytokines and MMPs stimulated by pristane-primed T cells, suggesting that the TLR3 signaling pathway induced by T cells actually caused the FLS activation. However, the questions how T cells upregulate TLR3 expression in FLSs, and whether TLR3 induction is due to cell-cell interaction or to T cell-derived soluble factor stimulation, were still equivocal. Thus, we utilized the T-cell conditioned medium instead of T cells to be incubated with the FLSs and found that FLSs with pristane-primed T-cell conditioned medium showed strong induction of TLR3 expression and increased production of cytokines and MMPs. The increased secretion of IFN-β and MMPs, but not IL-6, in FLSs was blocked by TLR3-specific RNAi. The above *in vitro *data suggested that the stimulation of soluble factors derived from T cells might be the main cause of TLR3 upregulation, which subsequently activated FLSs, although we still could not rule out the role of cell contact. IL-6 upregulation, which could be induced by a variety of stimuli, seemed not to depend on TLR3, suggesting that IL-6 might be regulated by other mechanisms.

The pristane-primed arthritogenic T cells which are used to transfer arthritis could secrete IFN-γ and TNF-α with Con A stimulation *in vitro*, and pretreatment of recipient rats with either anti-IFN-γ or a recombinant TNF-α receptor before transfer, ameliorated arthritis development [38]. Other cytokines are also considered to be involved in arthritis, however, and biological medicines targeting them (such as IL-1, IL-6 and IL-17) were developed in different clinical trials [39]. Thus, the question of which key cytokines participate in mediating local inflammation in joints is still interesting. In our rat experiments, we comprehensively used inflammatory factors to stimulate FLSs from RA patients to confirm the universality of TLR3 induction in the human system. All chosen factors, including proinflammatory cytokines and ligands of TLRs, which had been reported to be involved in RA successfully induced the upregulation of TLR3 expression. With poly(I:C) treatment, we observed an enhanced increase in TLR3 expression in RA FLSs compared with OA FLSs, suggesting that TLR3 signaling in RA FLSs has a lower activation threshold. These findings validate that in RA, not only in PIA, TLR3 signaling mediates the FLS activation induced by inflammatory stimulation.

## Conclusions

In summary, TLR3 in the synovium of PIA rats displayed early and persistent overexpression at the initiation and development stages of arthritis. TLR3 was localized in FLSs, and activation of the TLR3 signaling pathway *in vivo *could aggravate arthritis. FLSs were activated and made functional by the T cell-derived inflammatory mediator via TLR3 signaling in arthritis. Thus, TLR3 was confirmed to be an important mediator to bridge T cells and FLSs in arthritis. These data provide a very useful experimental example to increase understanding of the initiation and development of synovial inflammation systemically and dynamically, and also provide an interesting target for RA treatment.

## Abbreviations

DMEM: Dulbecco's modified Eagle's medium; FLS: fibroblast-like synoviocyte; H & E: hematoxylin and eosin; IFN: interferon; IgG: immunoglobulin G; IL: interleukin; LPS: lipopolysaccharide; MMP: matrix metalloproteinase; NF-κB: nuclear factor κB; NS: normal saline; OA: osteoarthritis; PBS: phosphate-buffered saline; PCR: polymerase chain reaction; PGN: peptidoglycan; PIA: pristane-induced arthritis; poly(I:C): polyinosinic:polycytidylic acid; RA: rheumatoid arthritis; RNAi: RNA interference; shRNA: short-hairpin RNA; TLR: Toll-like receptor; TNF: tumor necrosis factor.

## Competing interests

The authors declare that they have no financial or commercial conflicts of interest.

## Authors' contributions

WZ, LM, RH and SL conceived and designed the experiments. WZ and LM performed the experiments and analyzed the data. CJ, XH and WH performed some experiments. PX and HD collected samples. WZ, LM, RH and SL held extensive scientific discussions regarding this study and wrote the manuscript. All authors read and approved the final manuscript.

## Supplementary Material

Additional file 1 **Regulation of TLR expression in rat FLSs co-cultured with pristane primed T cells**. Rat FLSs were co-cultured with pristane primed or control T cells, and TLR1, TLR2 and TLR4 through TLR9 mRNA expression in FLSs was measured 24 hours after co-culture with series cell ratios of 5:1, 1:1, 1:5 and 1:10. Their expression in FLSs co-cultured with pristane-primed T cells was compared to that co-cultured with control T cells. Data are presented as means ± SEM of three replicated determinations from three independent experiments. Levels of significance were calculated by using Student's *t*-test (*P < 0.05). Click here for file

## References

[B1] Firestein GS Evolving concepts of rheumatoid arthritis Nature 2003 15 356 361 10.1038/nature01661 12748655

[B2] Feldmann M Brennan FM Maini RN Rheumatoid arthritis Cell 1996 85 307 310 10.1016/S0092-8674(00)81109-5 8616886

[B3] Müller-Ladner U Ospelt C Gay S Distler O Pap T Cells of the synovium in rheumatoid arthritis: synovial fibroblasts Arthritis Res Ther 2007 9 223 10.1186/ar2337 18177509 PMC2246247

[B4] Smith RS Smith TJ Blieden TM Phipps RP Fibroblasts as sentinel cells: synthesis of chemokines and regulation of inflammation Am J Pathol 1997 151 317 322 9250144 PMC1858004

[B5] Ospelt C Kyburz D Pierer M Seibl R Kurowska M Distler O Neidhart M Muller-Ladner U Pap T Gay RE Gay S Toll-like receptors in rheumatoid arthritis joint destruction mediated by two distinct pathways Ann Rheum Dis 2004 63 Suppl 2 ii90 ii91 15479881 10.1136/ard.2004.028324 PMC1766768

[B6] O'Neill LA Primer: Toll-like receptor signaling pathways-What do rheumatologists need to know? Nat Clin Pract Rheumatol 2008 4 319 327 10.1038/ncprheum0802 18446139

[B7] Barton GM Medzhitov R Toll-like receptor signaling pathways Science 2003 300 1524 1525 10.1126/science.1085536 12791976

[B8] Iwasaki A Medzhitov R Toll-like receptor control of the adaptive immune responses Nat Immunol 2004 5 987 995 10.1038/ni1112 15454922

[B9] Seibl R Birchler T Loeliger S Hossle JP Gay RE Saurenmann T Michel BA Seger RA Gay S Lauener RP Expression and regulation of Toll-like receptor 2 in rheumatoid arthritis synovium Am J Pathol 2003 162 1221 1227 10.1016/S0002-9440(10)63918-1 12651614 PMC1851232

[B10] Ospelt C Brentano F Rengel Y Stanczyk J Kolling C Tak PP Gay RE Gay S Kyburz D Overexpression of Toll-like receptors 3 and 4 in synovial tissue from patients with early rheumatoid arthritis: Toll-like receptor expression in early and longstanding arthritis Arthritis Rheum 2008 58 3684 3692 10.1002/art.24140 19035519

[B11] Roelofs MF Joosten LAB Abdollahi-Roodsaz S van Lieshout AWT Sprong T van den Hoogen FH van den Berg WB Radstake T The expression of Toll-like receptors 3 and 7 in rheumatoid arthritis synovium is increased and costimulation of Toll-like receptors 3, 4, and 7/8 results in synergistic cytokine production by dendritic cells Arthritis Rheum 2005 52 2313 2322 10.1002/art.21278 16052591

[B12] Huang QQ Ma YY Adebayo A Pope RM Increased macrophage activation mediated through Toll-like receptors in rheumatoid arthritis Arthritis Rheum 2007 56 2192 2201 10.1002/art.22707 17599732

[B13] van der Heijden IM Wilbrink B Tchetverikov I Schrijver IA Schouls LM Hazenberg MP Breedveld FC Tak PP Presence of bacterial DNA and bacterial peptidoglycans in joints of patients with rheumatoid arthritis and other arthritides Arthritis Rheum 2000 43 593 598 10.1002/1529-0131(200003)43:3<593::AID-ANR16>3.0.CO;2-1 10728753

[B14] Roelofs MF Boelens WC Joosten LAB Abdollahi-Roodsaz S Geurts J Wunderink LU Schreurs BW van den Berg WB Radstake T Identification of small heat shock protein B8 (HSP22) as a novel TLR4 ligand and potential involvement in the pathogenesis of rheumatoid arthritis J Immunol 2006 176 7021 7027 16709864 10.4049/jimmunol.176.11.7021

[B15] Brentano F Schorr O Gay RE Kyburz D RNA released from necrotic synovial fluid cells activates rheumatoid arthritis synovial fibroblasts via Toll-like receptor 3 Arthritis Rheum 2005 52 2656 2665 10.1002/art.21273 16142732

[B16] Liu ZQ Deng GM Foster S Tarkowski A Staphylococcal peptidoglycans induce arthritis Arthritis Res 2001 3 375 380 10.1186/ar330 11714392 PMC64849

[B17] Deng GM Nilsson IM Verdrengh M Collins LV Tarkowski A Intra-articularly localized bacterial DNA containing CpG motifs induces arthritis Nat Med 1999 5 702 705 10.1038/9554 10371511

[B18] Iwahashi M Yamamura M Aita T Okamoto A Ueno A Ogawa N Akashi S Miyake K Godowski PJ Makino H Expression of Toll-like receptor 2 on CD16+ blood monocytes and synovial tissue macrophages in rheumatoid arthritis Arthritis Rheum 2004 50 1457 1467 10.1002/art.20219 15146415

[B19] Jung YO Cho ML Kang CM Jhun JY Park JS Oh HJ Min JK Park SH Kim HY Toll-like receptor 2 and 4 combination engagement upregulate IL-15 synergistically in human rheumatoid synovial fibroblasts Immunol Lett 2007 109 21 27 10.1016/j.imlet.2006.12.006 17289161

[B20] Pierer M Rethage J Seibl R Lauener R Brentano F Wagner U Hantzschel H Michel BA Gay RE Gay S Kyburz D Chemokine secretion of rheumatoid arthritis synovial fibroblasts stimulated by Toll-like receptor 2 ligands J Immunol 2004 172 1256 1265 14707104 10.4049/jimmunol.172.2.1256

[B21] Cho ML Ju JH Kim HR Oh HJ Kang CM Jhun JY Lee SY Park MK Min JK Park SH Lee SH Kim HY Toll-like receptor 2 ligand mediates the upregulation of angiogenic factor, vascular endothelial growth factor and interleukin-8 CXCL8 in human rheumatoid synovial fibroblasts Immunol Lett 2007 108 121 128 10.1016/j.imlet.2006.11.005 17182109

[B22] Kim KW Cho ML Lee SH Oh HJ Kang CM Ju JH Min SY Cho YG Park SH Kim HY Human rheumatoid synovial fibroblasts promote osteoclastogenic activity by activating RANKL via TLR-2 and TLR-4 activation Immunol Lett 2007 110 54 64 10.1016/j.imlet.2007.03.004 17467812

[B23] Marshak-Rothstein A Toll-like receptors in systemic autoimmune disease Nat Rev Immunol 2006 6 823 835 10.1038/nri1957 17063184 PMC7097510

[B24] Meng L Zhu W Jiang C He X Hou W Zheng F Holmdahl R Lu S Toll-like receptor 3 upregulation in macrophages participates in the initiation and maintenance of pristane-induced arthritis in rats Arthritis Res Ther 2010 12 R103 10.1186/ar3034 20500834 PMC2911891

[B25] Vingsbo C Sahlstrand P Brun JG Jonsson R Saxne T Holmdahl R Pristane-induced arthritis in rats: a new model for rheumatoid arthritis with a chronic disease course influenced by both major histocompatibility complex and non-major histocompatibility complex genes Am J Pathol 1996 149 1675 1683 8909256 PMC1865278

[B26] Yaron M Baratz M Yaron I Zor U Acute induction of joint inflammation in the rat by poly I. poly C Inflammation 1979 3 243 251 10.1007/BF00914181 478595

[B27] Laragione T Brenner M Mello A Symons M Gulko PS The arthritis severity locus *Cia5d *is a novel genetic regulator of the invasive properties of synovial fibroblasts Arthritis Rheum 2008 58 2296 2306 10.1002/art.23610 18668563 PMC2714698

[B28] Volin MV Huynh N Klosowska K Chong KK Woods JM Fractalkine is a novel chemoattractant for rheumatoid arthritis fibroblast-like synoviocyte signaling through MAP kinases and Akt Arthritis Rheum 2007 56 2512 2522 10.1002/art.22806 17665439

[B29] Julius MH Simpson E Herzenberg LA Rapid method for isolation of functional thymus-derived murine lymphocytes Eur J Immunol 1973 3 645 649 10.1002/eji.1830031011 4587740

[B30] Arnett FC Edworthy SM Bloch DA McShane DJ Fries JF Cooper NS Healey LA Kaplan SR Liang MH Luthra HS Medsger TA Jr Mitchell DM Neustadt DH Pinals RS Schaller JG Sharp JT Wilder RL Hunder GG The American Rheumatism Association 1987 Revised Criteria for the Classification of Rheumatoid Arthritis Arthritis Rheum 1988 31 315 324 10.1002/art.1780310302 3358796

[B31] Heinz S Haehnel V Karaghiosoff M Schwarzfischer L Müller M Krause SW Rehli M Species-specific regulation of Toll-like receptor 3 genes in men and mice J Biol Chem 2003 278 21502 21509 10.1074/jbc.M301476200 12672806

[B32] Kim KW Cho ML Oh HJ Kim HR Kang CM Heo YM Lee SH Kim HY TLR-3 enhances osteoclastogenesis through upregulation of RANKL expression from fibroblast-like synoviocytes in patients with rheumatoid arthritis Immunol Lett 2009 124 9 17 10.1016/j.imlet.2009.02.006 19446344

[B33] Rückert R Brandt K Ernst M Marienfeld K Csernok E Metzler C Budagian V Bulanova E Paus R Bulfone-Paus S Interleukin-15 stimulates macrophages to activate CD4^+ ^T cells: a role in the pathogenesis of rheumatoid arthritis? Immunology 2009 126 63 73 10.1111/j.1365-2567.2008.02878.x 18557790 PMC2632696

[B34] Hoffmann MH Tuncel J Skriner K Tohidast-Akrad M Türk B Pinol-Roma S Serre G Schett G Smolen JS Holmdahl R Steiner G The rheumatoid arthritis-associated autoantigen hnRNP-A2 (RA33) is a major stimulator of autoimmunity in rats with pristane-induced arthritis J Immunol 2007 179 7568 7576 18025202 10.4049/jimmunol.179.11.7568

[B35] Sakaguchi N Takahashi T Hata H Nomura T Tagami T Yamazaki S Sakihama T Matsurtani T Negishi I Nakatsuru S Sakaguchi S Altered thymic T-cell selection due to a mutation of the ZAP-70 gene causes autoimmune arthritis in mice Nature 2003 426 454 460 10.1038/nature02119 14647385

[B36] Janossy G Panayi G Duke O Bofill M Poulter LW Goldstein G Rheumatoid arthritis: a disease of t-lymphocyte/macrophage immunoregulation Lancet 1981 2 839 842 6116956 10.1016/s0140-6736(81)91107-7

[B37] Klareskog L Forsum U Scheynius A Kabelitz D Wigzell H Evidence in support of a self-perpetuating HLA-DR-dependent delayed-type cell reaction in rheumatoid arthritis Proc Natl Acad Sci USA 1982 79 3632 3636 10.1073/pnas.79.11.3632 6980416 PMC346477

[B38] Holmberg J Tuncel J Yamada H Lu S Olofsson P Holmdahl R Pristane, a non-antigenic adjuvant, induces MHC class II-restricted, arthritogenic T cells in the rat J Immunol 2006 176 1172 1179 16394006 10.4049/jimmunol.176.2.1172

[B39] McInnes IB Schett G Cytokines in the pathogenesis of rheumatoid arthritis Nat Rev Immunol 2007 7 429 442 10.1038/nri2094 17525752

