# Supplementary figures and images for: Arthritis is associated with T-cell-induced upregulation of Toll-like receptor 3 on synovial fibroblasts
Source: Arthritis Res Ther. 2011 Jun 27;13(3):R103. doi: 10.1186/ar3384 (PMC3218918; doi:10.1186/ar3384)

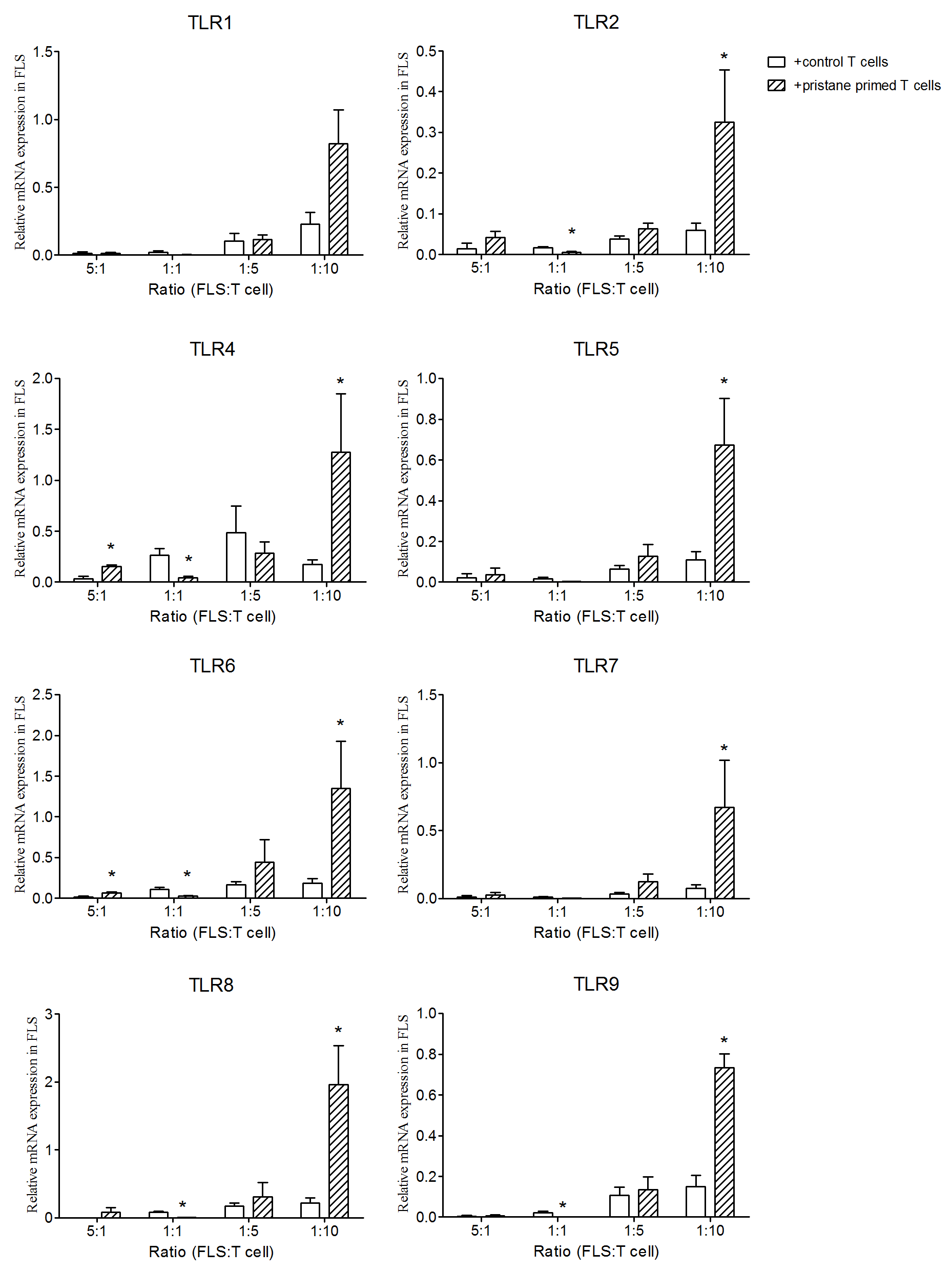

Supplement: Additional file 1 — Regulation of TLR expression in rat FLSs co-cultured with pristane primed T cells. Rat FLSs were co-cultured with pristane primed or control T cells, and TLR1, TLR2 and TLR4 through TLR9 mRNA expression in FLSs was measured 24 hours after co-culture with series cell ratios of 5:1, 1:1, 1:5 and 1:10. Their expression in FLSs co-cultured with pristane-primed T cells was compared to that co-cultured with control T cells. Data are presented as means ± SEM of three replicated determinations from three independent experiments. Levels of significance were calculated by using Student's t-test (*P < 0.05). [file ar3384-S1.TIFF]
